# Brain-type natriuretic peptide is a useful biomarker of cardiovascular disease and predictor of cardiac-related mortality in chimpanzees (*Pan troglodytes*)

**DOI:** 10.2460/ajvr.24.09.0287

**Published:** 2024-12-23

**Authors:** Angela M. Achorn, Carolyn L. Hodo, Martha E. Hensel, Elizabeth R. Magden, Stephanie J. Buchl, Charla L. Jones, Elizabeth A. Piatt, William D. Hopkins

**Affiliations:** Michale E. Keeling Center for Comparative Medicine and Research, MD Anderson Cancer Center, University of Texas, Bastrop, TX

**Keywords:** cardiovascular disease, brain natriuretic peptide, primates, chimpanzees, great apes

## Abstract

**OBJECTIVE:**

To validate the use of brain-type natriuretic peptide (BNP) for detecting and monitoring cardiac dysfunction in captive chimpanzees (*Pan troglodytes*).

**METHODS:**

We analyzed cross-sectional (N = 175) and longitudinal (N = 76) BNP, echocardiogram, ECG, and pathology data from living and deceased captive chimpanzees to examine age and sex effects and to assess the usefulness of BNP for detecting cardiovascular disease and predicting mortality. The study period was from July 2010 through October 2024.

**RESULTS:**

Brain-type natriuretic peptide values were significantly associated with age, and males exhibited significantly higher BNP values than females. Brain-type natriuretic peptide values were significantly heritable, with over half of variation in BNP being attributable to additive genetic effects. Chimpanzees with more severe cardiac scores based on echocardiograms and ECGs had significantly higher BNP values. Among 50 deceased chimpanzees, those with initial BNP values > 100 pg/mL were significantly more likely to have a cardiac-related causes of death than those with values < 100 pg/mL.

**CONCLUSIONS:**

Brain-type natriuretic peptide values reflected cardiac scores, and lower BNP values were associated with increased survival rates. Importantly, BNP values over a clinical threshold of 100 pg/mL were a significant predictor of cardiac death.

**CLINICAL RELEVANCE:**

Cardiovascular disease is a leading cause of morbidity and mortality in great apes, and sudden cardiac death with few to no preceding clinical signs is common in chimpanzees. A biomarker for occult heart disease could help identify individuals requiring monitoring or intervention. Brain-type natriuretic peptide is a useful biomarker for heart disease in chimpanzees, which has important clinical relevance for facilities that house captive chimpanzees.

Natriuretic peptides (NPs) are a family of hormones/paracrine factors that regulate blood pressure and volume, ventricular hypertrophy, pulmonary hypertension, fat metabolism, and long bone growth.^1–3^ Natriuretic peptides can exert effects through a number of actions, such as vasodilation, metabolism of glucose and lipids, natriuresis, lipid mobilization and oxidation, and improved insulin sensitivity.^4^ They can also exert paracrine and autocrine actions directly on the heart, which can help prevent arrhythmias, cardiomyopathies, hypertrophy, fibrosis, and the development and progression of heart failure.^4^ In humans, known NPs include atrial NP (ANP), B-type NP (or “brain NP”; BNP), and C-type NP (CNP).^5^ Atrial NP is secreted from the cardiac atria, and its main function is using hormone and paracrine signaling to decrease blood pressure and cardiac hypertrophy.^6^ Brain-type NP is secreted from the cardiac ventricles, and it mainly serves to reduce ventricular fibrosis.^6^ Research suggests that ANP and BNP inhibit cardiac fibrosis by modulating renin-angiotensin-aldosterone system signaling.^7^ Unlike ANP and BNP, which are both expressed in the heart, CNP is primarily secreted from the CNS but is also found in endothelial cells and chondrocytes.^8^ C-type NP has been shown to stimulate long bone growth and to cause dilation of arteries and veins, though the vasodilation and blood pressure reductions elicited by CNP are believed to be less significant than those triggered by ANP.^6,9^

Brain-type NP in particular is used as a biomarker to diagnose and assess the severity of heart failure since BNP levels have been shown to increase in humans with cardiac disease due to myocardial stress and volume overload.^2,10,11^ Indeed, BNP is an extremely effective clinical measure for assessing heart failure in humans. According to the FDA and the European Society of Cardiology, the BNP clinical reference range for humans is < 100 pg/mL.^12–14^ One study^15^ involving 1,586 humans reported that at a cutoff of ≥ 100 pg/mL, diagnostic accuracy was 81.2%, sensitivity was 90%, specificity was 73%, positive predictive value was 75%, negative predictive value was 90%, and the positive likelihood ratio was 3.4. Another study^13^ with 1,050 humans found that at a cutoff of 100 pg/mL, BNP demonstrated 82% sensitivity and 99% specificity for distinguishing control patients and patients with congestive heart failure. Based on these reports, it is generally accepted that a clinically normal BNP level (< 100 pg/mL) indicates a low probability of heart failure, whereas an elevated BNP level signals the need for additional cardiac tests.^2,12–16^ As in humans, cardiovascular disease is one of the leading causes of morbidity and mortality in the nonhuman great apes.^17–19^ However, while other apes are physiologically, genetically, and behaviorally similar to humans due to our phylogenetic proximity, the underlying cause and presentation of heart disease differs between species.^17,20^ One major difference is that coronary artery disease, a significant pathology in humans, is not found in other great apes.^20^ Additionally, in humans, congestive heart failure is the most common presentation of cardiovascular disease, whereas nonhuman apes more often suffer sudden cardiac death with few preceding clinical signs. In nonhuman apes, histology after death often reveals interstitial myocardial fibrosis or fibrosing cardiomyopathy, though the etiology and mechanism of the fibrosis formation requires further investigation.^20–23^ Interstitial myocardial fibrosis occurs when collagen fibers spread diffusely through the cardiac muscle fibers, and it can be triggered by stimuli such as pressure or volume overload.^18^ Fibrosing cardiomyopathy, a term used almost exclusively for nonhuman apes, refers to severe interstitial myocardial fibrosis replacing and separating myofibers within all heart chambers, particularly in the left ventricle, interventricular septum, and subvalvular regions.^24^ Interstitial myocardial fibrosis can be triggered by stimuli such as pressure or volume overload or can occur as a repair mechanism following myocardial damage.^18^ There is still much to learn about the etiology, determinants, and risk factors of cardiovascular disease in great apes. In chimpanzees specifically, arrhythmias (particularly ventricular ectopy) may signal the development of cardiomyopathy, and cardiac murmurs may signal valvular disease; however, in most cases, there appear to be few other clinical signs.^25^ Given the “silent” nature of sudden cardiac death in chimpanzees, a biomarker for detecting occult heart disease could help identify individuals requiring enhanced monitoring or medical intervention.

While the clinical utility of BNP has been well demonstrated in humans, assessing BNP as a biomarker to diagnose or monitor cardiac disease in nonhuman primates has been understudied. A recent study by Pai et al^26^ assessed ANP and BNP levels, echocardiographs, and ECGs in cynomolgus macaques (*Macaca fascicularis*) to establish NP reference values and to evaluate their effectiveness as indicators of cardiac disease in this species. These authors found that ANP and BNP levels were significantly higher in monkeys with valvular disease compared to healthy monkeys and that ANP and BNP levels increased with the progression of valvular disease, suggesting that these NPs are useful indicators of cardiovascular disease in cynomolgus monkeys. Brain-type NP has also been examined in captive western lowland gorillas (*Gorilla gorilla gorilla*) by Murray et al^21^ as part of the Great Ape Heart Project. These authors evaluated records from zoo-housed gorillas to assess relationships between BNP and cardiovascular disease. They found that elevated BNP levels correlated with visible echocardiographic abnormalities and with reported clinical signs consistent with cardiovascular decompensation, such as coughing, lethargy, exercise intolerance, social withdrawal, dyspnea, or grabbing at their chest. Finally, in chimpanzees (*Pan troglodytes*), BNP was 1 of 4 measures Ely et al^22^ evaluated as biomarkers for identifying and monitoring chimpanzees at risk of cardiovascular disease or sudden cardiac death. Of the 4 biomarkers (BNP, complete lipid panel, C-reactive protein, and cardiac troponin I), they determined that BNP and possibly cardiac troponin I were useful diagnostic biomarkers for cardiovascular disease. However, as this study included surrogate clinical endpoints rather than actual mortality, they recommend more extensive studies incorporating mortality rates and postmortem confirmation of myocardial lesions.

The goal of this present study was to validate the use of BNP for detecting and monitoring cardiac dysfunction in captive chimpanzees. We include cross-sectional data from a relatively large cohort of chimpanzees—both living and deceased, with and without reported cardiovascular disease—to further assess the usefulness of BNP for detecting cardiovascular disease and predicting mortality. Further, for deceased chimpanzees, we incorporate data from pathology reports, such as whether heart conditions had been identified as the proximate cause of death and whether myocardial lesions were present at postmortem or histopathological examination. Finally, in a subset of chimpanzees, we examined longitudinal measures of BNP as a means of assessing age-related changes in BNP levels. In short, validating BNP as a reliable biomarker of heart disease that can be assessed from blood samples rather than through cardiac imaging could facilitate earlier diagnosis, which would hopefully improve health outcomes and quality of life for these animals.

We also estimated the heritability of BNP values in our chimpanzee colony. Research with humans from the Framingham Heart Study has shown that having a parent with cardiovascular disease increases one’s chances of cardiac events independent of traditional risk factors.^27^ Further, Wang et al^28^ estimated the genetic heritability of N-terminal pro-atrial natriuretic peptide and BNP in nearly 2,000 Framingham Heart Study participants and found that both log N-terminal pro-atrial natriuretic peptide (*h*^2^ = 0.44; *P* < .0001) and log BNP (*h*^2^ = 0.35; *P* < .0001) were significantly heritable after controlling for age, sex, and other clinical and echocardiographic variables. These findings indicate that a substantial proportion of variation in NP levels was attributable to additive genetic effects.^28^ Given the crucial role that NP levels play in responding to cardiac overload, it is important to understand how additive genetic factors contribute to interindividual variation in BNP levels among chimpanzees as well.

## Methods

### Subjects

All chimpanzees (*P troglodytes*) were housed at the Chimpanzee Care Center at The University of Texas MD Anderson Cancer Center. Chimpanzees were housed in social groups of 3 to 6 individuals with 24-hour access to indoor/outdoor enclosures (except during cleaning) with bedding, climbing structures, and daily environmental enrichment. Chimpanzees were fed a commercial primate diet (Teklad Global Diets #7195 from Inotiv) and fresh produce, with daily foraging opportunities and ad libitum access to water. For this study, there was both a cross-sectional and longitudinal analysis of BNP levels. The cross-sectional analyses included 175 chimpanzees, 74 males and 101 females (75 deceased chimpanzees and 100 still living), with at least 1 BNP data point. This cohort ranged from 9 to 51 years of age (mean, 28.5; SD, 9.9) when the first samples were submitted for BNP analysis. Within this sample of 175 chimpanzees, 76 subjects were included in longitudinal analyses as they had additional BNP data points in the archived health records at least 3 years after the initial one. This longitudinal cohort was comprised of 36 males and 40 females, ranging from 10 to 49 years of age at the first sampling point and 13 to 56 years at the next sampling point. The number of years between samples ranged from 3 to 13. The study period during which we obtained BNP, echocardiogram, ECG, or pathology data was from July 7, 2010, through October 31, 2024. All experimental procedures were carried out according to the NIH guidelines for animal research and were approved by the IACUC at the University of Texas MD Anderson Cancer Center.

### Clinical and cardiac assessment

During physical examinations, a veterinary cardiologist performed echocardiograms and ECGs on a subset of 91 chimpanzees. Measurements taken during echocardiograms included the size of all 4 heart chambers, left ventricular chamber size, right ventricular wall thickness, interventricular septum thickness, and left ventricular free wall (posterior wall) thickness in systole and diastole, and contractility indices (see Supplementary Material for a full list of parameters examined during echocardiograms). Based on echocardiograms and ECGs, the veterinary cardiologist assigned cardiac scores of A, B, C, or D to the chimpanzees. These cardiac scores, which were based on the American Heart Association and the American College of Cardiology’s 4-stage classification of heart failure^29^ and modified by Dr. Charla Jones, grade the clinical severity of cardiac disease. Briefly, cardiac score A indicates no structural heart disease. Cardiac score B indicates early structural heart disease without clinical signs of heart failure. Cardiac score C indicates current or past clinical signs of heart failure. Cardiac score D represents end-stage heart disease with clinical signs of heart failure.^30^ See Table 1 for full descriptions of cardiac score criteria. For BNP analyses, EDTA whole-blood samples were collected during physical examinations. Plasma was separated and stored at −80 °C until being shipped on dry ice to the Baylor Scott & White Medical Center Reference Laboratory. Plasma samples were typically shipped the day they were collected or within 1 week of collection if there were plans to collect additional samples in the following days. At the reference laboratory, the ARCHITECT BNP assay (Abbott) was used for the quantitative determination of human B-type NP.

### Pathology

Postmortem examinations were done following standard necropsy procedures consistent with the Great Ape Heart Project Recommended Cardiac Necropsy Prosection Guide.^31^ Briefly, external measurements were taken, and structural changes to the heart were assessed, including an examination of the left and right atrioventricular valves, aortic valve, and pulmonic valve. The presence of any secondary lesions associated with cardiopulmonary failure were noted, such as chronic passive congestion of the liver, pulmonary hemorrhage and edema, tri-cavitary transudate effusions, SC edema, and mediastinal lymphadenopathy. The heart was weighed, and the thickness of the left and right ventricular free wall and the interventricular septum was measured. Sections of all 4 chambers and the interventricular septum were fixed in 10% neutral-buffered formalin and processed routinely for histopathology. Following gross and histopathological examinations, lesions and final diagnoses and conclusions regarding cause of death were recorded in individual necropsy reports. For this study, a board-certified veterinary pathologist retrospectively reviewed all the necropsy reports for the study subjects and recorded whether cardiac factors were determined to be the proximate cause of death (or of the clinical signs leading to euthanasia). Heart disease was considered the proximate cause of death if no other significant disease was identified and if secondary lesions of heart disease, such as chronic passive congestion of the liver, pulmonary edema and hemorrhage, valvular endocardiosis with fibroelastosis (“jet lesions”) or mural thromboembolism, were noted. Additionally, the heart lesion severity was confirmed based on histologic evidence of cardiomyocyte degeneration and loss with interstitial fibrosis. We set whether or not cardiac factors were the proximate cause of death as a binary variable (1 = cardiac proximate COD; 0 = noncardiac proximate COD). For analyses involving mortality rates and pathological findings, we used October 31, 2024, as the cutoff point for whether subjects were alive or deceased, and we used their first BNP measure as the predictor variable to assess whether early BNP values predict subsequent mortality.

### Statistical analyses

#### Age, bodyweight, and sex effects on BNP

**Figure 1—:**
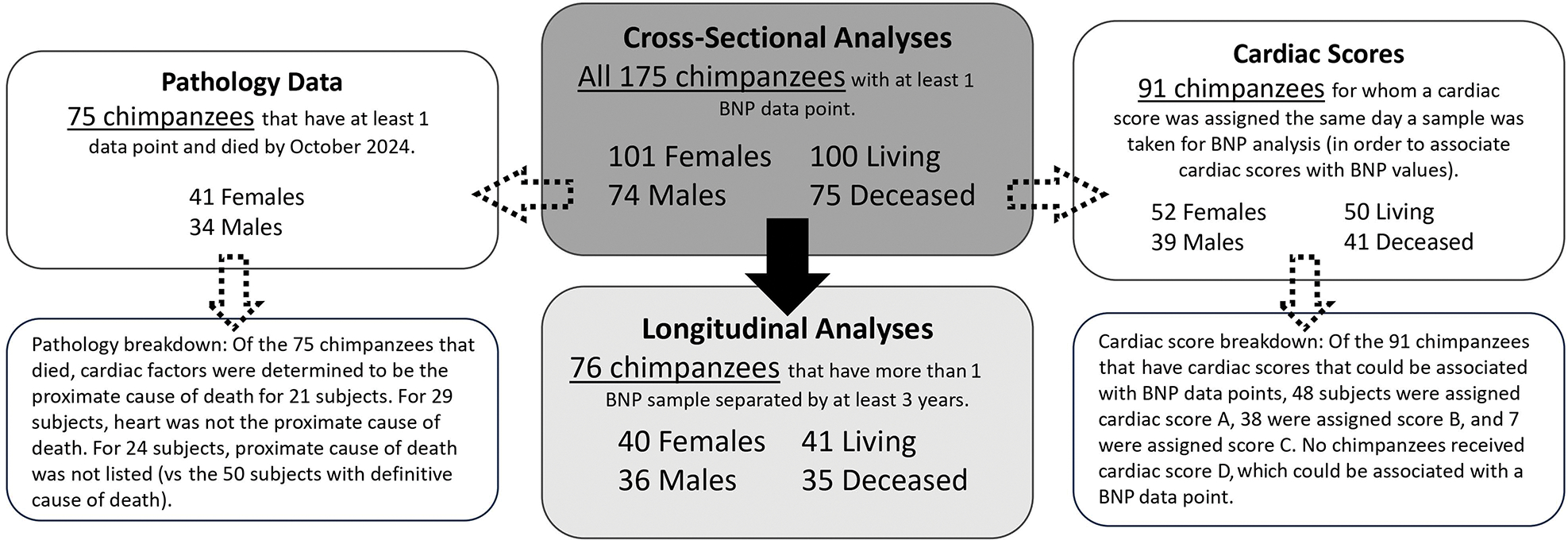
The goal of this study was to validate the use of brain-type natriuretic peptide (BNP) for detecting and monitoring cardiac dysfunction in captive chimpanzees (*Pan troglodytes*). We analyzed cross-sectional (N = 175) and longitudinal (N = 76) BNP, echocardiogram, ECG, and pathology data from living and deceased captive chimpanzees to examine age and sex effects and to assess the usefulness of BNP for detecting cardiovascular disease and predicting mortality. The study period was from July 2010 through October 2024. Different statistical analyses required different subgroupings of chimpanzees. This figure shows the demographic breakdown and criteria for inclusion for each subgroup.

An initial box plot analysis identified that the BNP values were highly variable, with several outliers evident in the distribution of scores. Additionally, a Shapiro-Wilk test indicated that neither the cross-sectional data nor the longitudinal data were normally distributed (*P* < .001). Therefore, we opted to use nonparametric statistics for most analyses. Spearman rank order correlations were used to test for significant associations between BNP values and (1) age and (2) body weight at the time of the sample. Mann-Whitney *U* tests and a Kruskal-Wallis test were used to compare BNP values between groups of apes (eg, living vs deceased; subjects that died of cardiac disease vs ones with no clinical signs of heart disease). Because different statistical analyses required different subgroupings of chimpanzees, Figure 1 shows the demographic breakdown and criteria for inclusion for each subgroup.

#### Cross-sectional approach

For the cross-sectional approach specifically (N = 175), to assess whether higher initial BNP values correlated with subsequent mortality in the chimpanzees, we calculated a difference score between the chimpanzees’ age at death and their age at the time of sample collection. Higher scores indicated a longer period of time between their age when the BNP sample was collected and their age at death. We correlated this difference score with their BNP values. Additionally, to further examine whether initial BNP concentrations predicted cardiac death, we also used Kaplan-Meier curves to test for differences between 2 groups (N = 75 deceased chimpanzees): subjects with initial BNP concentrations > 100 pg/mL and subjects with initial concentrations < 100 pg/mL.

#### Longitudinal approach

As an alternative to the cross-sectional analytic approach, we also performed longitudinal analyses. Within the full cohort of 175 chimpanzees, there were 76 chimpanzees (41 alive and 35 deceased) that had BNP data points that were separated by at least 3 years. We computed BNP difference scores for time points 1 and 2 for the BNP values, body weights, and ages of each chimpanzee. In terms of the scale of measurement, time point 1 data points were subtracted from time point 2. Thus, higher difference scores for BNP values, bodyweight, and age values reflected an increase in those measures over time. We used a Spearman rank order correlation to test for significant associations between the difference in BNP scores and changes in either body weight or age. We then rank ordered the BNP difference scores for the sample and compared the rank-ordered difference values between the living and deceased individuals for the entire sample as well as within male and female cohorts.

#### Cardiac health, pathology, and BNP

Χ^2^ tests of independence were used to test for associations between dichotomous variables. Specifically, we used a χ^2^ to test if, among a sample of 50 deceased chimpanzees with known causes of death, those with initial BNP values > 100 pg/mL were more likely to have cardiac factors listed as their proximate cause of death than those with initial BNP values < 100 pg/mL. Here, the number of years between initial BNP and death ranged from 0.00 to 13.57 years (mean, 5.32 years; SE, 0.41), and the goal of this analysis was to test whether early BNP values predicted subsequent cardiac death. Because other studies^12–14,22^ have suggested a cutoff value of 150 pg/mL in gorillas^21^ and 163 pg/mL in chimpanzees, we ran 2 additional χ^2^ tests using 150 pg/mL and 163 pg/mL as the cutoff points to see how these findings compare to a cutoff of 100 pg/mL. To test whether the most recently reported BNP values were associated with pathology findings, we also ran χ^2^ tests using deceased subjects’ last BNP score rather than their first. For these analyses, the duration between subjects’ last BNP score and time of death ranged from 0.0 to 10.34 years (mean, 1.74 years; SE, 0.35). Finally, to compare the usefulness of BNP versus cardiac scores for predicting cardiac death in chimpanzees, we also used a χ^2^ test to determine if, among chimpanzees that have died, cardiac scores assigned at the same time as subjects’ initial BNP values were associated with proximate cause of death (cardiac vs other causes).

### Heritability

Consistent with previous studies^32–34^ that have utilized pedigree information from captive nonhuman primate populations, we used the software program Sequential Oligogenic Linkage Analysis Routines (SOLAR-Eclipse 8.3.1 developed by Maryland Psychiatric Research Center)^35^ to estimate heritability in BNP values based on pedigree information from our chimpanzee colony. Prior to heritability analyses, the phenotype (BNP values) was normalized (due to high kurtosis) using an inverse normal transformation function within Sequential Oligogenic Linkage Analysis Routines. Covariates included age, sex, rearing history, sex*age, sex*rearing history, and age*rearing history. Rearing history refers to whether the chimpanzees were dam reared, nursery reared, or wild caught. We include it as a covariate since rearing has been shown to affect health outcomes in primates.^36^

## Results

### Heritability, age, bodyweight, and sex effects on BNP

Spearman rank order correlations revealed significant associations between BNP values and age (*r* = + 0.269; *P* < .001) but not between BNP values and body weight (*r* = + 0.019; *P* = .845). A Mann-Whitney *U* test revealed significant differences in BNP values between males and females (*P* = .009). Males (N = 74; mean, 143.21; mean rank, 99.77; median, 72.5; Quartile 1 [Q1], 44.8; Q3, 112.0) had higher BNP values, on average, than females (N = 101; mean, 87.50; mean rank, 79.38; median, 52.0; Q1, 33.7; Q3, 94.5). Brain-type NP values were found to be significantly heritable (*h*^2^ = 0.54; SE, 0.168; *P* = .0003). Significant covariates for BNP values in the heritability analysis included age (*P* = .016) and sex (*P* = .019), and the proportion of variance accounted for by these covariates was 0.147.

### Mortality and BNP

#### Cross-sectional analysis

**Figure 2—:**
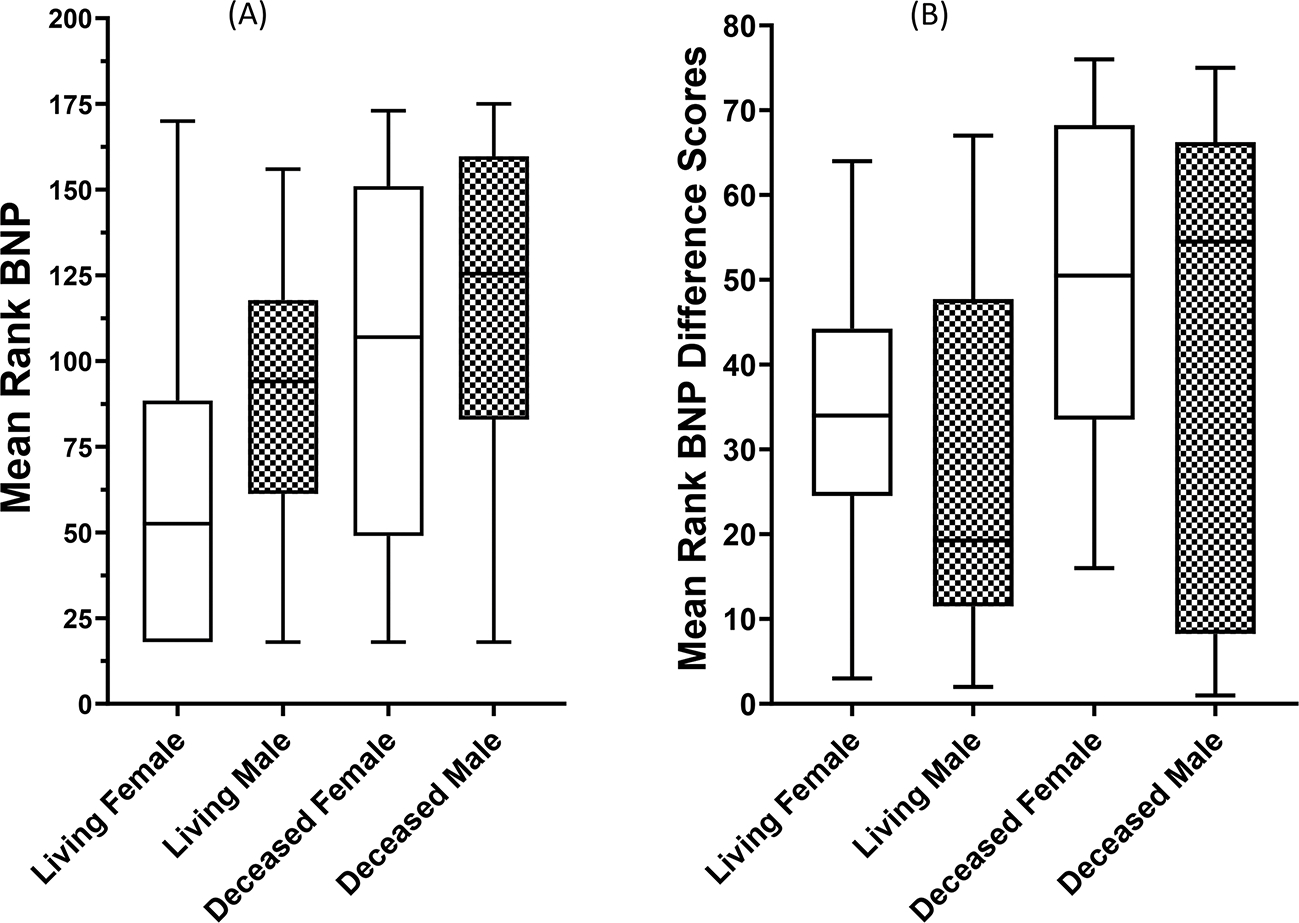
Cross-sectional analysis: among the sample of 175 chimpanzees described in Figure 1, a Mann-Whitney *U* test for the overall sample comparing BNP values in living (N = 101; mean, 59.44; mean rank, 72.43; median, 44.80; quartile 1 (Q1), 25.00; Q3, 73.70) and deceased (N = 74; mean, 179.21; mean rank, 109.26; median, 85.55; Q1, 42.10; Q3, 179.00) chimpanzees was significant (*P* < .001). This was also the case within female (*P* = .001) and male (*P* < .001) cohorts. As can be seen in (A), for both males and females, deceased chimpanzees had higher BNP values than those that were still living as of October 2024. Longitudinal analyses: Within the full sample of 175 chimpanzees, there were 76 chimpanzees (41 alive, 35 deceased) that had BNP data points that were separated by at least 3 years. We computed BNP difference scores for time points 1 and 2 for the BNP values (thus, higher BNP difference scores reflect an increase over time). Mann-Whitney *U* tests revealed significant differences in BNP difference scores between the living and deceased individuals for the entire sample (*P* = .001), with deceased chimpanzees having higher BNP difference scores than those still living. Separate analyses of the female and male samples revealed significant differences between living and deceased difference values for females (*P* = .022), and for males, the difference approached significance (*P* = .059). As seen in (B), these analyses indicate that increases in BNP values between the 2 sampling time points were much greater for chimpanzees that subsequently died compared to those still living. For (A) and (B), the solid lines in the boxes depict median values, the top and bottom of the box depict upper and lower quartile values, and the whiskers depict maximum and minimum data values.

**Figure 3—:**
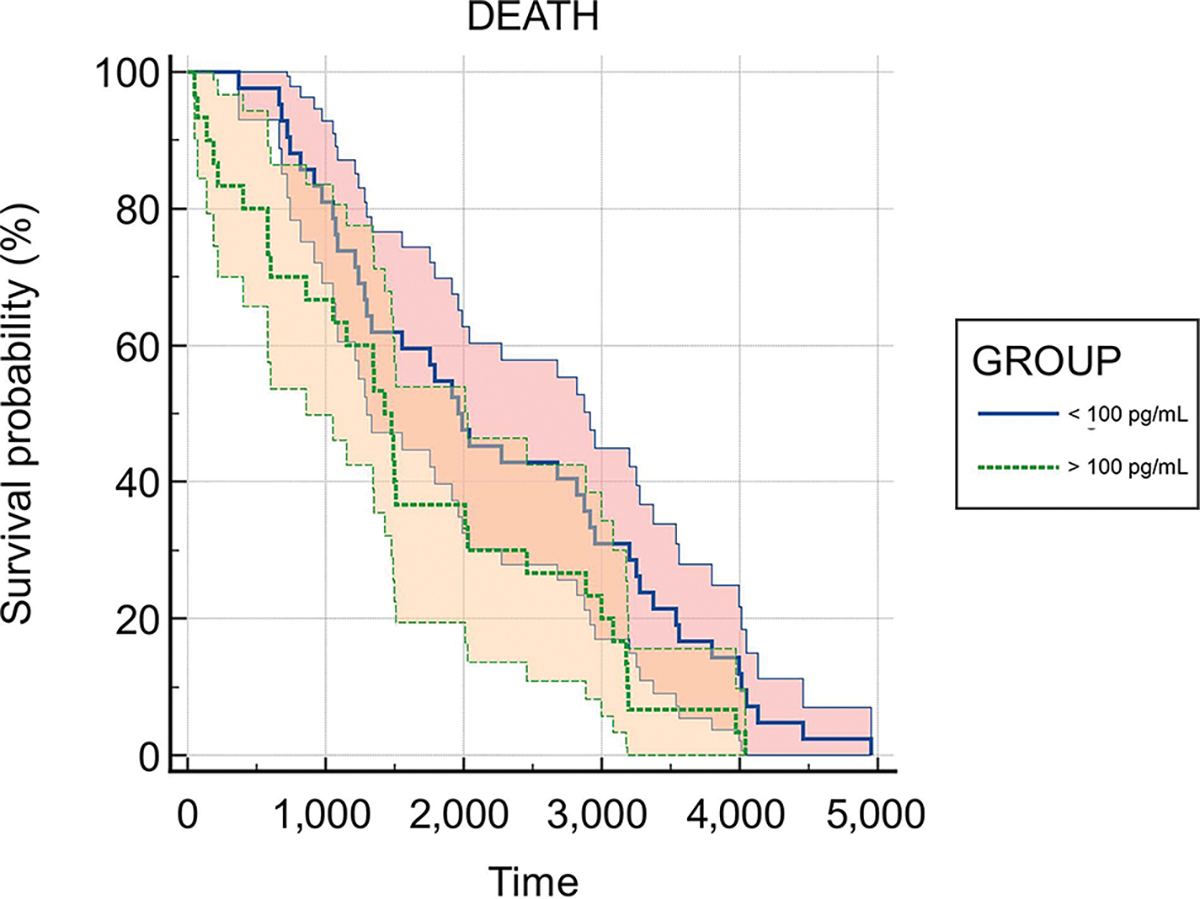
Among the sample of chimpanzees described in Figure 1, there are 75 chimpanzees (41 females, 34 males) that died by October 2024. For these 75 deceased subjects, Kaplan-Meier survival analysis showing time (in number of days) between initial BNP data points and death for 2 groups: those with an initial BNP value < 100 pg/mL (N = 42, blue solid line) and those with an initial BNP value > 100 pg/mL (N = 33, green dotted line). The graph includes 95% CIs. A steeper slope (ie, BNP > 100 pg/mL) indicates a higher event rate (death rate) and therefore a worse survival prognosis. A less steep slope (ie, BNP < 100 pg/mL) indicates a lower event rate and therefore a better survival prognosis. A comparison of these survival curves (log-rank test) revealed significant differences in survival between the 2 groups (χ^2^ = 4.5953; degrees of freedom, 1; *P* = .0321).

The Mann-Whitney *U* test for the overall sample comparing BNP values in living (N = 101; mean, 59.44; mean rank, 72.43; median, 44.80; Q1, 25.00; Q3, 73.70) and deceased (N = 74; mean, 179.21; mean rank, 109.26; median, 85.55; Q1, 42.10; Q3, 179.00) chimpanzees was significant (*P* < .001). This was also the case within the female (*P* = .001) and male (*P* < .001) cohorts. As shown in Figure 2, for both males and females and for the overall sample, deceased chimpanzees had higher BNP values than those that were still living as of October 2024. A Mann-Whitney *U* test comparing BNP values between (1) chimpanzees that showed no clinical signs of heart disease on echocardiograms or ECGs and that are still alive and (2) chimpanzees that died from cardiac disease revealed a significant difference between groups (*P* < .001). Subjects that died from cardiac disease (N = 21; mean, 385.94; mean rank, 54.19; median, 175.10; Q1, 123.35; Q3, 275.75) had larger BNP values, on average, than subjects that did not show signs of clinical disease (cardiac score A; N = 48; mean, 68.58; mean rank, 26.6; median, 53.45; Q1, 37.00; Q3, 87.10). A Spearman rank order correlation between age difference scores (ie, age at death minus age at sample collection) and BNP values revealed a modest negative correlation value (*r* = − 0.382; *P* = .002), indicating that lower BNP values at the sample date were associated with increasing survival rate (measured by number of years between sample collection and death). A comparison of Kaplan-Meier survival curves (log-rank test) revealed significant differences in survival between chimpanzees with initial BNP values > 100 pg/mL (33) and those with initial BNP values < 100 pg/mL (N = 42). Here, chimpanzees with initial BNP values < 100 pg/mL experienced increased survival (χ^2^ = 4.5953; degrees of freedom, 1; *P* = .0321; Figure 3).

#### Longitudinal analyses

Spearman rank order correlations revealed no significant associations between the difference in BNP scores and changes in either body weight (*r* = + 0.144; *P* = .213) or age (*r* = − 0.002; *P* = .990). Thus, increases in BNP values across time points were neither greater nor smaller as a function of the difference in age time between sample collections or changes in bodyweight. However, the Mann-Whitney *U* test comparing the rank-ordered difference values between the living and deceased individuals for the entire sample was significant (*P* = .001), with deceased chimpanzees (N = 35; mean, 312.82; mean rank, 47.37; median, 60.60; Q1, 25.70; Q3, 250.40) having higher rank-ordered BNP difference values than those still living (N = 41; mean, 21.69; mean rank, 30.93; median, 22.00; Q1, −15.85; Q3, 39.70). Separate analyses of the female and male samples revealed significant differences between living and deceased difference values for females (*P* = .022), and for males, the difference approached significance (*P* = .059). These analyses indicate that increases in BNP values between the 2 sampling time points were much greater for chimpanzees that subsequently died compared to those still living (Figure 2).

### Cardiac health, pathology, and BNP

**Figure 4—:**
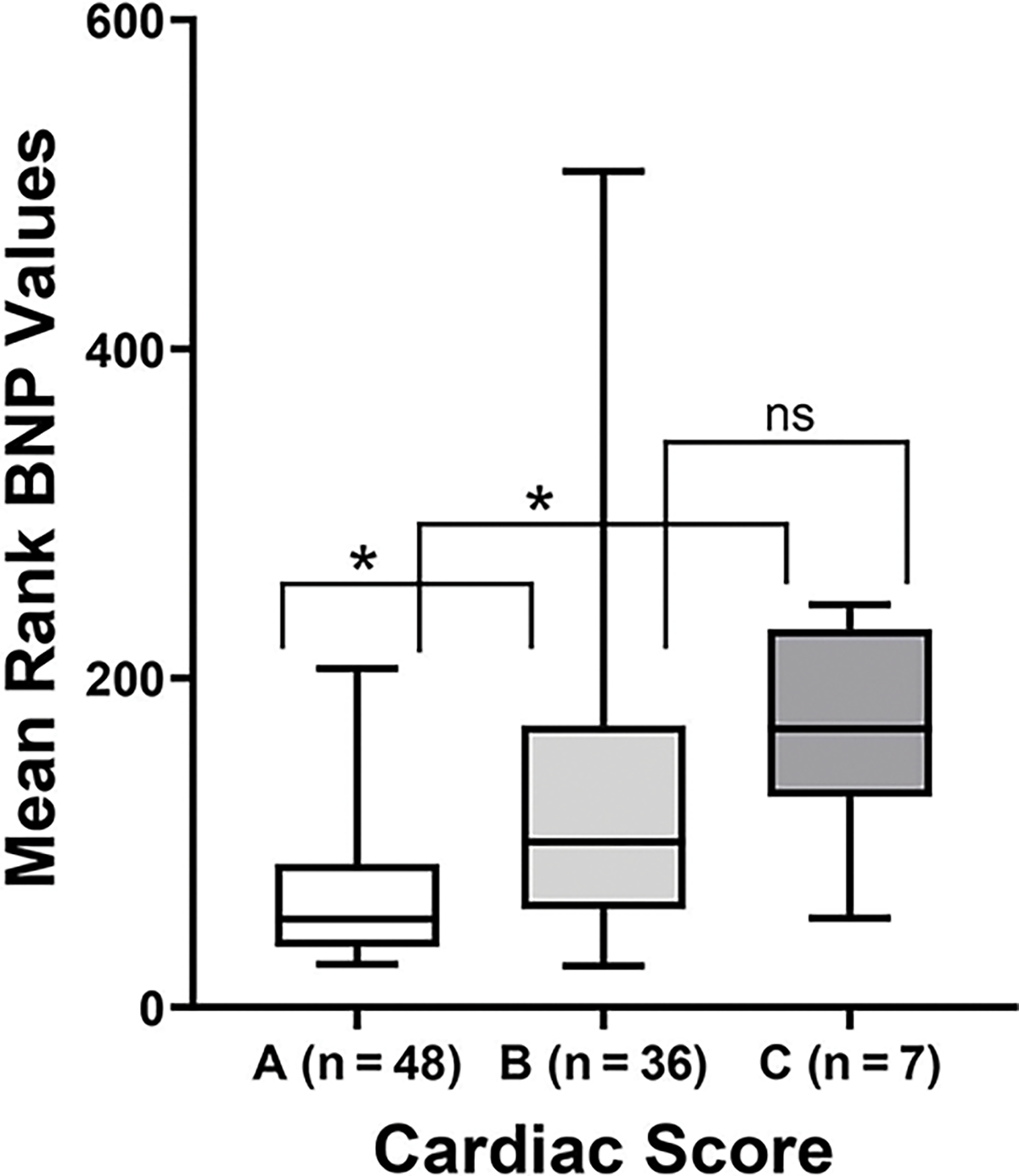
Among the sample of chimpanzees described in Figure 1, there are 91 subjects (50 living, 41 deceased) for whom a cardiac score was assigned the same day a sample was taken for BNP analysis. Thus, we were able to associate cardiac scores with BNP values for these subjects to assess whether BNP values significantly differed between cardiac scores. A Kruskal-Wallis test revealed significant differences in BNP values between cardiac score categories (*P* < .001). Chimpanzees with a cardiac score of B (N = 36; mean, 132.57; mean rank, 54.61) or C (N = 7; mean, 172.60; mean rank, 71.86) exhibited higher BNP values, on average, than chimpanzees with a cardiac score of A (N = 48; mean, 68.58; mean rank, 35.77). In our sample, no chimpanzees received cardiac score D in association with a BNP sample. This figure shows mean rank BNP values between cardiac scores—the solid lines in the boxes depict median values, the top and bottom of the box depict upper and lower quartile values, and the whiskers depict maximum and minimum data values. *Significance at *P* < .05. ns = Not significant.

**Figure 5—:**
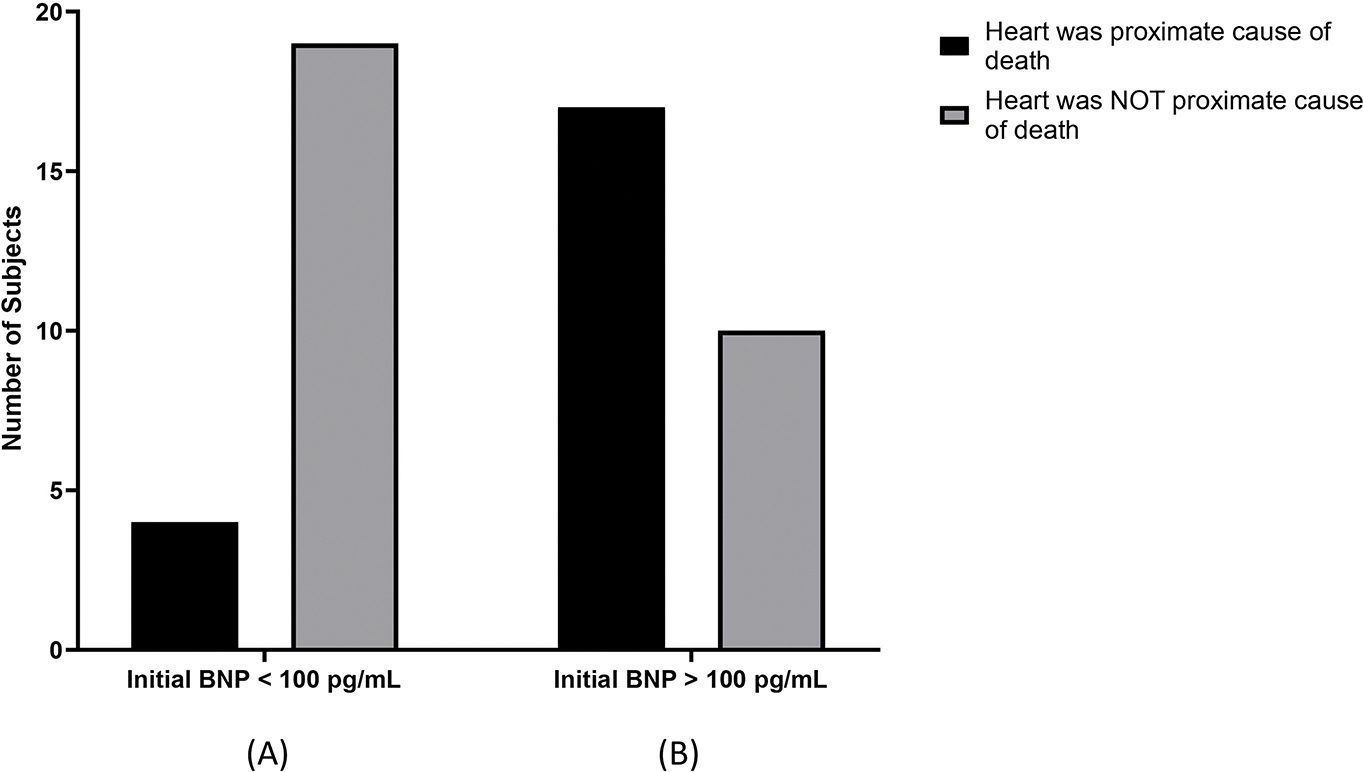
A χ^2^ test of independence was used to assess if, among a sample of 50 deceased chimpanzees with known causes of death (described in Figure 1), those with initial BNP values > 100 pg/mL were more likely to have cardiac factors listed as their proximate cause of death than those with initial BNP values < 100 pg/mL. Here, the number of years between initial BNP and death ranged from 0.00 to 13.57 years (mean, 5.32 years; SE, 0.41), and the goal of this analysis was to test whether early BNP values predicted subsequent cardiac death. The χ^2^ test was statistically significant (χ^2^ = 10.588; N = 50; *P* = .001). Chimpanzees that had an initial BNP value > 100 pg/mL were significantly more likely to have cardiac factors listed as their proximate cause of death than those that had initial BNP scores < 100 pg/mL. Indeed, 17 of 21 chimpanzees that apparently died from heart-related causes (81%; depicted by the black bars on the graph) had an initial BNP value > 100 pg/mL.

Among the 91 chimpanzees that received cardiac scores, a Kruskal-Wallis test revealed significant differences in BNP values between cardiac score categories (*P* < .001). As shown in Figure 4, chimpanzees with a cardiac score of B (N = 36; mean, 132.57; mean rank, 54.61) or C (N = 7; mean, 172.60; mean rank, 71.86) exhibited higher BNP values, on average, than chimpanzees with a cardiac score of A (N = 48; mean, 68.58; mean rank, 35.77). In our sample, no chimpanzees received a cardiac score of D in association with a BNP data point. Using a sample of 50 deceased chimpanzees for whom cause of death was known (with BNP values ranging from 25.0 to 2,773.4; mean, 176.6; SEM, 40.8), a χ^2^ test^12,22^ examining the likelihood of dying from heart-related issues based on BNP values > or < 100 pg/mL was statistically significant (χ^2^ = 10.588; N = 50; *P* = .001). Specifically, those that had an initial BNP value > 100 pg/mL were significantly more likely to have cardiac factors listed as their proximate cause of death than those that had initial BNP scores < 100 pg/mL. Indeed, 17 of 21 chimpanzees that apparently died from heart-related causes (81%) had an initial BNP value > 100 pg/mL (Figure 5) from samples collected from 2009 through 2013. Because other studies^21,22^ of BNP in African apes have suggested a reference value of 150 pg/mL in gorillas and 163 pg/mL in chimpanzees, we ran an additional χ^2^ test using 150 pg/mL as the cutoff point (no chimpanzees had initial BNP values between 150 to 163 pg/mL). While the results were still statistically significant (χ^2^ = 7.025, N = 50; *P* = .008), only 12 of 21 chimpanzees that died from heart-related causes (57.1%) had BNP values > 150 pg/mL. A χ^2^ test using subjects’ last BNP values rather than their first revealed that among chimpanzees that have died, a last BNP value > 100 pg/mL was associated with the presence of heart lesions at necropsy (χ^2^ = 4.216; N = 39; *P* = .040) and with cardiac factors being the proximate cause of death (χ^2^ = 7.657; N = 39; *P* = .006). Finally, among the 29 deceased chimpanzees that received cardiac scores at the same time as their first BNP values, a χ^2^ test examining cardiac scores and proximate cause of death (cardiac vs other causes) revealed no significant association (χ^2^ = 4.999; *P* = .082). According to this analysis, 2 of 15 chimps with cardiac scores that died from heart-related causes (13.3%) had been assigned a cardiac score of A, 11 of 15 (73.3%) had a score of B, and 2 of 15 (13.3%) had a score of C.

## Discussion

Cardiovascular disease is a leading cause of morbidity and mortality in captive chimpanzees; therefore, it is extremely valuable to have a biomarker of heart disease to help identify at-risk individuals. The primary goal of this study was to assess the potential utility of BNP as a biomarker to detect or monitor cardiac abnormalities. Our results indicate that BNP values reliably reflect cardiac scores derived from echocardiograms and ECGs. Chimpanzees that were assigned cardiac scores of B (early structural heart disease with no clinical signs of heart failure) or C (current or past clinical signs of heart failure) by a veterinary cardiologist exhibited significantly higher BNP values than those that were assigned cardiac score A (no structural heart disease). Furthermore, higher BNP values were significantly associated with mortality outcomes. The first evidence of this comes from a retrospective comparison of differences in BNP values between 2 sampling time points for deceased and living chimpanzees in 2024. This comparison revealed that for both males and females, chimpanzees that died in the years since sampling exhibited much greater increases in BNP values between time points than those that are still alive. Next, for the subset of chimpanzees that did die, there was a negative association between BNP values and survival, such that chimpanzees with higher BNP values tended to have fewer years between when the sample was collected and when they died. Finally, when comparing chimpanzees that died from heart disease to those that did not, a statistically significant majority of individuals with heart-related mortality had BNP values > 100 pg/mL, the threshold of clinical significance in humans.

Regarding the clinical cutoff point at which BNP values can detect cardiac abnormalities, human medical texts recommend 100 pg/mL based on evidence that a BNP value < 100 pg/mL indicates a low probability of heart failure.^13^ The present study assessed if this threshold of clinical significance is also a useful benchmark for chimpanzees and found that it is. A comparison of Kaplan-Meier survival curves revealed significant differences in survival between chimpanzees with initial BNP values > 100 pg/mL and those with initial BNP values < 100 pg/mL, wherein chimpanzees with initial BNP values < 100 pg/mL experienced increased survival. Furthermore, chimpanzees with an initial BNP value > 100 pg/mL were significantly more likely to have cardiac factors as their proximate cause of death than those that had initial BNP values < 100 pg/mL, and 17 of 21 chimpanzees that died from heart-related causes (81%) had an initial BNP value > 100 pg/mL. Our results suggest that BNP values predicted cardiac death more reliably than cardiac scores derived from echocardiograms and ECGs; however, it is worth noting that cardiac scores in this study were collected over 10 years before death for some subjects. Additionally, because interstitial myocardial fibrosis cannot always be evaluated on an echocardiogram, the degree of interstitial myocardial fibrosis is often not reflected in the cardiac score even though it significantly contributes to chimpanzee cardiac death. This further demonstrates the usefulness of BNP values for detecting cardiac abnormalities that may not be observed through imaging alone.

Heritability estimates indicate that a substantial amount of variation in BNP values (54.1%) is attributable to additive genetic effects. To our knowledge, no previous studies have examined the heritability of NPs in great apes, but research has shown BNP values to be significantly heritable in humans.^28^ A strong genetic basis for BNP values is pertinent clinical information given the extensive pedigree knowledge that exists for captive chimpanzee populations.

Finally, the results revealed significant sex differences in BNP values, with males exhibiting higher BNP mean ranks than females. Sex was also found to be a significant covariate in BNP heritability estimates. These observed sex effects are perhaps unsurprising since heart disease is more common in male chimpanzees and is more often the proximate cause of death in male chimpanzees than in females.^37,38^ In humans, sex differences in cardiovascular disease are sometimes attributed to the protective properties of estrogen on the heart, the expression of certain Y chromosome genes that influence blood pressure and stress response, and other genetic and environmental factors.^38^ This may be true for chimpanzees as well.

This study had a few limitations. Because this was a retrospective study using a longitudinal dataset, we did not have all of the information needed to perform gold standard tests of sensitivity and specificity for BNP. Additionally, though we compared BNP values between different cardiac score categories—which were used to classify the clinical severity of heart disease and are based on echocardiogram and ECG parameters—we do not link BNP to specific metrics, such as heart chamber measurements, ejection fraction, concentric remodeling, or filling pressures. This would be valuable for future studies to investigate. Lastly, there are other conditions besides heart disease that could result in mild elevations of BNP values, such as obesity, renal disease, or older age. It is therefore possible that the BNP values reported in this study are influenced by noncardiac factors, though elevations from these other conditions would presumably be slight. Despite these limitations, the study offers sufficient evidence that BNP is associated with heart dysfunction and mortality in chimpanzees.

This is not the first study to recommend using BNP to detect and monitor cardiac abnormalities in great apes. Ely et al^22^ and Murray et al^21^ have demonstrated the potential usefulness of BNP to detect heart disease in chimpanzees and gorillas, respectively. However, this study analyzed data cross-sectionally and longitudinally and incorporated pathology data from necropsies to further assess BNP’s specific role in both morbidity and mortality in a population of captive chimpanzees. These findings support and build upon previous studies by showing that BNP values reliably reflect cardiac scores derived from echocardiograms and ECGs, that lower BNP values are associated with increased survival rates, and that higher BNP values (over a cutoff point of 100 pg/mL) are a significant predictor of dying from heart-related conditions.^21,22^ It therefore appears that BNP is a clinically useful biomarker for detecting and monitoring heart disease in chimpanzees. These results have clinical relevance for facilities that house captive chimpanzees wherein the minimally invasive assessment of BNP values along with other bloodwork can help determine which animals may be at risk of heart disease and therefore may require further cardiac evaluation, monitoring, or treatment.

## Supplementary Material

Supplemental_Material

Supplementary materials are posted online at the journal website: avmajournals.avma.org.

## Figures and Tables

**Table 1—:** Cardiac score classification for heart disease in chimpanzees (based on the American Heart Association and the American College of Cardiology’s 4-stage classification of heart failure, modified by Dr. Charla L. Jones).

Cardiac score	Full description

A	Chimpanzees that have not developed structural heart changes or clinical signs of heart disease (eg, those with diabetes; systemic hypertension; coronary disease without prior infarct; mild arrhythmias, such as occasional single unifocal premature ventricular contractions or single premature atrial contractions; first- or mild second-degree atrioventricular block; mild age-related or congenital heart valve changes; slight-to-mild valve insufficiency; or stenosis with no heart chamber dilation).
B	Chimpanzees with structural heart disease or worsening arrhythmias that have not yet developed clinical signs of heart failure (eg, multifocal and/or frequent premature ventricular contractions, atrial flutter or fibrillation or paroxysmal atrial tachycardia, moderate-to-severe second-degree atrioventricular block, reduced ejection fraction, left ventricular hypertrophy, chamber enlargement, or moderate-to-severe valve insufficiency).
C	Chimpanzees that have developed clinical signs of heart disease (eg, decreased activity tolerance, episodes of syncope or collapse, signs of left- or right-sided congestive heart failure, ascites, peripheral limb edema, coughing, tachypnea, dyspnea, cough, or cyanosis). Subjects responding to treatment may have resolution of clinical heart signs and improved heart structure and function but may remain in this score category.
D	Chimpanzees with refractory heart failure.

## References

[1] Potter LR, Abbey-Hosch S, Dickey DM. Natriuretic peptides, their receptors, and cyclic guanosine monophosphate-dependent signaling functions. Endocr Rev. 2006;27(1):47–72. doi:10.1210/er.2005-0014 16291870

[2] Novack ML, Zubair M. Natriuretic peptide B type test. StatPearls. StatPearls Publishing; 2023. Accessed August 1, 2024. https://www.ncbi.nlm.nih.gov/books/NBK556136/32310596

[3] Daniels LB, Maisel AS. Natriuretic peptides. J Am Coll Cardiol. 2007;50(25):2357–2368. doi:10.1016/j.jacc.2007.09.021 18154959

[4] Sarzani R, Allevi M, Di Pentima C, Schiavi P, Spannella F, Giulietti F. Role of cardiac natriuretic peptides in heart structure and function. Int J Mol Sci. 2022;23(22):14415. doi:10.3390/ijms232214415 36430893 PMC9697447

[5] Nakagawa Y, Nishikimi T, Kuwahara K. Atrial and brain natriuretic peptides: hormones secreted from the heart. Peptides. 2019;111:18–25. doi:10.1016/j.peptides.2018.05.012 29859763

[6] Potter LR, Yoder AR, Flora DR, Antos LK, Dickey DM. Natriuretic peptides: their structures, receptors, physiologic functions and therapeutic applications. Handb Exp Pharmacol. 2009;191:341–366. doi:10.1007/978-3-540-68964-5_15 PMC4855512 19089336

[7] Kerkelä R, Ulvila J, Magga J. Natriuretic peptides in the regulation of cardiovascular physiology and metabolic events. J Am Heart Assoc. 2015;4(10):e002423. doi:10.1161/JAHA.115.002423 26508744 PMC4845118

[8] Moyes AJ, Hobbs AJ. C-type natriuretic peptide: a multifaceted paracrine regulator in the heart and vasculature. Int J Mol Sci. 2019;20(9):2281. doi:10.3390/ijms20092281 31072047 PMC6539462

[9] Nakagawa Y, Nishikimi T. CNP, the third natriuretic peptide: its biology and significance to the cardiovascular system. Biology. 2022;11(7):986. doi:10.3390/biology11070986 36101368 PMC9312265

[10] Koratala A, Kazory A. Natriuretic peptides as biomarkers for congestive states: the cardiorenal divergence. Dis Markers. 2017;2017:1454986. doi:10.1155/2017/1454986 28701807 PMC5494089

[11] Doust J, Lehman R, Glasziou P. The role of BNP testing in heart failure. Am Fam Physician. 2006;74(11):1893–1898. 17168346

[12] Pagana K, Pagana T, Pagana T. Mosby’s Diagnostic & Laboratory Test Reference. 14th ed. ed. Elsevier; 2019.

[13] Wieczorek SJ, Wu AHB, Christenson R, A rapid B-type natriuretic peptide assay accurately diagnoses left ventricular dysfunction and heart failure: a multicenter evaluation. Am Heart J. 2002;144(5):834–839. doi:10.1067/mhj.2002.125623 12422152

[14] Ponikowski P, Voors AA, Anker SD, 2016 ESC guidelines for the diagnosis and treatment of acute and chronic heart failure: The Task Force for the diagnosis and treatment of acute and chronic heart failure of the European Society of Cardiology (ESC). Developed with the special contribution of the Heart Failure Association (HFA) of the ESC. Eur J Heart Fail. 2016;18(8):891–975. doi:10.1002/ejhf.592 27207191

[15] McCullough PA, Nowak RM, McCord J, B-type natriuretic peptide and clinical judgment in emergency diagnosis of heart failure. Circulation. 2002;106(4):416–422. doi:10.1161/01.CIR.0000025242.79963.4C 12135939

[16] Maisel AS, Krishnaswamy P, Nowak RM, Rapid measurement of B-type natriuretic peptide in the emergency diagnosis of heart failure. N Engl J Med. 2002;347(3):161–167. doi:10.1056/NEJMoa020233 12124404

[17] Drane AL, Atencia R, Cooper S-M, Cardiac structure and function characterized across age groups and between sexes in healthy wild-born captive chimpanzees (Pan troglodytes) living in sanctuaries. Am J Vet Res. 2018;80(6):547–557. doi:10.2460/ajvr.80.6.547 31140849

[18] Lammey ML, Baskin GB, Gigliotti AP, Lee DR, Ely JJ, Sleeper MM. Interstitial myocardial fibrosis in a captive chimpanzee (Pan troglodytes) population. Comp Med. 2008;58(4):389–394. 18724782 PMC2706041

[19] van Zijll Langhout M, Wolters M, Horvath KM, Clinical signs, diagnostics and successful treatment of a myocarditis in an adult chimpanzee (Pan troglodytes). J Med Primatol. 2017;46(5):263–266. doi:10.1111/jmp.12273 28523858

[20] Varki N, Anderson D, Herndon J, Heart disease is common in humans and chimpanzees, but is caused by different pathological processes. Evol Appl. 2009;2(1):101–112. doi:10.1111/j.1752-4571.2008.00064.x 25567850 PMC3352420

[21] Murray S, Kishbaugh J, Haye KL, Diagnosing cardiovascular disease in western lowland gorillas (Gorilla Gorilla Gorilla) with brain natriuretic peptide. PLoS One. 2019;14(3):e0214101. doi:10.1371/journal.pone.0214101 30889217 PMC6424555

[22] Ely JJ, Zavaskis T, Lammey ML, Sleeper MM, Lee DR. Association of brain-type natriuretic protein and cardiac troponin I with incipient cardiovascular disease in chimpanzees (Pan troglodytes). Comp Med. 2011;61(2):163–169. 21535928 PMC3079819

[23] Strong V, Moittié S, Sheppard MN, Idiopathic myocardial fibrosis in captive chimpanzees (Pan troglodytes). Vet Pathol. 2020;57(1):183–191. doi:10.1177/0300985819879442 31640487

[24] Baldessari A, Snyder J, Ahrens J, Murnane R. Fatal myocardial fibrosis in an aged chimpanzee (Pan troglodytes). Pathobiol Aging Age Relat Dis. 2013;3. 10.3402/pba.v3i0.21073 PMC3679521 23762500

[25] Doane CJ, Lee DR, Sleeper MM. Electrocardiogram abnormalities in captive chimpanzees (Pan troglodytes). Comp Med. 2006;56(6):512–518. 17219782

[26] Pai C, Nakayama S, Ito-Fujishiro Y, Usefulness of cardiac hormones for evaluating valvular disease in cynomolgus monkeys (Macaca fascicularis). J Vet Med Sci. 2021;83(4):716–723. doi:10.1292/jvms.20-0606 33692223 PMC8111363

[27] Lloyd-Jones DM, Nam B-H, D’Agostino S, Ralph B, Parental cardiovascular disease as a risk factor for cardiovascular disease in middle-aged adults: a prospective study of parents and offspring. J Am Med Assoc. 2004;291(18):2204–2211. doi:10.1001/jama.291.18.2204 15138242

[28] Wang TJ, Larson MG, Levy D, Heritability and genetic linkage of plasma natriuretic peptide levels. Circulation. 2003;108(1):13–16. doi:10.1161/01.CIR.0000081657.83724.A7 12821537

[29] Yancy CW, Jessup M, Bozkurt B, 2013 ACCF/AHA guideline for the management of heart failure: a report of the American College of Cardiology Foundation/American Heart Association Task Force on practice guidelines. Circulation. 2013;128(16):e240–e327. doi:10.1161/CIR.0b013e31829e8776 23741058

[30] Great Ape Heart Project Recommended Cardiac Necropsy Prosection Guide. Great Ape Heart Project. Accessed August 3, 2024. https://greatapeheartproject.org/wp-content/uploads/2011/11/gahp-recommended-cardiac-necropsy-guide.pdf

[31] Fears SC, Melega WP, Service SK, Identifying heritable brain phenotypes in an extended pedigree of vervet monkeys. J Neurosci. 2009;29(9):2867. doi:10.1523/JNEUROSCI.5153-08.2009 19261882 PMC2716293

[32] Hopkins WD, Russell JL, Schaeffer J. Chimpanzee intelligence is heritable. Curr Biol. 2014;24(14):1649–1652. doi:10.1016/j.cub.2014.05.076 25017206 PMC4108509

[33] Rogers J, Shelton SE, Shelledy W, Garcia R, Kalin NH. Genetic influences on behavioral inhibition and anxiety in juvenile rhesus macaques. Genes Brain Behav. 2008;7(4):463–469. doi:10.1111/j.1601-183X.2007.00381.x 18045243 PMC2785008

[34] Almasy L, Blangero J. Multipoint quantitative-trait linkage analysis in general pedigrees. Am J Hum Genet. 1998;62(5):1198–1211. doi:10.1086/301844 9545414 PMC1377101

[35] Conti G, Hansman C, Heckman JJ, Novak MFX, Ruggiero A, Suomi SJ. Primate evidence on the late health effects of early-life adversity. Proc Natl Acad Sci USA. 2012;109(23):8866–8871. doi:10.1073/pnas.1205340109 22615410 PMC3384158

[36] Seiler BM, Dick EJ Jr., Guardado-Mendoza R, Spontaneous heart disease in the adult chimpanzee (Pan troglodytes). J Med Primatol. 2009;38(1):51–58. doi:10.1111/j.1600-0684.2008.00307.x 18671767 PMC2933140

[37] Lammey ML, Lee DR, Ely JJ, Sleeper MM. Sudden cardiac death in 13 captive chimpanzees (Pan troglodytes). J Med Primatol. 2008;37(1):39–43. 10.1111/j.1600-0684.2007.00260.x 18269527

[38] Leinwand LA. Sex is a potent modifier of the cardiovascular system. J Clin Invest. 2003;112(3):302–307. doi:10.1172/JCI19429 12897194 PMC166308

