# Supplementary material for: Brain-type natriuretic peptide is a useful biomarker of cardiovascular disease and predictor of cardiac-related mortality in chimpanzees (Pan troglodytes)
Source: Am J Vet Res. Author manuscript; Available in PMC 2026 Feb 26. (PMC12936050; doi:10.2460/ajvr.24.09.0287)
Supplement: Supplemental_Material [file NIHMS2136337-supplement-Supplemental_Material.pdf]

Supplementary Material. List of parameters examined during echocardiograms.

- Left atrial diameter
- Aortic root diameter
- Left atrial diameter divided by aortic root diameter
- Aortic cusp separation
- Left ventricle ejection time
- Right ventricle anterior wall thickness
- Right ventricle diameter
- Interventricular septum thickness – diastole
- Interventricular septum thickness – systole
- Interventricular septum thickness – percent change
- Left ventricular posterior wall thickness – diastole
- Left ventricular posterior wall thickness – systole
- Left ventricular posterior wall thickness – percent change
- Interventricular septum divided by left ventricular posterior wall
- Left ventricular diameter – diastole
- Left ventricular diameter – systole
- Left ventricular diameter – percent change
- Mean velocity of circumferential fiber shortening
- Left ventricular volume – diastole
- Left ventricular volume – systole
- Stroke volume
- Left ventricular ejection fraction
- Left ventricular fractional shortening
